# *Plasmodium falciparum* Molecular Chaperones: Guardians of the Malaria Parasite Proteome and Renovators of the Host Proteome

**DOI:** 10.3389/fcell.2022.921739

**Published:** 2022-05-16

**Authors:** Gregory L. Blatch

**Affiliations:** ^1^ The Vice Chancellery, The University of Notre Dame Australia, Fremantle, WA, Australia; ^2^ Biomedical Biotechnology Research Unit, Department of Biochemistry and Microbiology, Rhodes University, Grahamstown, South Africa; ^3^ Biomedical Research and Drug Discovery Research Group, Faculty of Health Sciences, Higher Colleges of Technology, Sharjah, United Arab Emirates

**Keywords:** heat shock proteins, molecular chaperones and co-chaperones, malaria parasite, parasitophorous vacuole, proteostasis

## Abstract

*Plasmodium falciparum* is a unicellular protozoan parasite and causative agent of the most severe form of malaria in humans. The malaria parasite has had to develop sophisticated mechanisms to preserve its proteome under the changing stressful conditions it confronts, particularly when it invades host erythrocytes. Heat shock proteins, especially those that function as molecular chaperones, play a key role in protein homeostasis (proteostasis) of *P. falciparum*. Soon after invading erythrocytes, the malaria parasite exports a large number of proteins including chaperones, which are responsible for remodeling the infected erythrocyte to enable its survival and pathogenesis. The infected host cell has parasite-resident and erythrocyte-resident chaperones, which appear to play a vital role in the folding and functioning of *P. falciparum* proteins and potentially host proteins. This review critiques the current understanding of how the major chaperones, particularly the Hsp70 and Hsp40 (or J domain proteins, JDPs) families, contribute to proteostasis of the malaria parasite-infected erythrocytes.

## Introduction

The deadliest human malaria parasite, *Plasmodium falciparum,* has a reduced genome, and yet appears to have dedicated a significant proportion of its genes (∼2%) to molecular chaperones (Sargeant et al., 2006), the guardians of protein folding. This suggests that the structural integrity of the proteome is an important aspect of the survival of the malaria parasite. Interestingly, an unusually high proportion of *P. falciparum* proteins (24–30%) are rich in asparagine (N) and glutamine (Q), particularly poly-N repeats (Singh et al., 2004; Pallarès et al., 2018), which have been found to have a tendancy to aggregate (Halfmann et al., 2011). Furthermore, a key phase in the pathology of malaria, is the invasion of host erythrocytes by the parasite, which it completely remodels by exporting over 400 parasite proteins, including a substantial proportion (∼5%) of molecular chaperones (Cortés et al., 2020). This massive renovation of the host cell potentially requires unique protein folding pathways involving both parasite and host molecular chaperones (Pesce and Blatch, 2014; Gabriela et al., 2022). This review will critique the evidence indicating that heat shock proteins serving as molecular chaperones, especially Hsp70 and Hsp40 (also called J domain proteins, JDPs) families, are highly adapted to maintaining the structural and functional integrity of the proteomes of the parasite and potentially the host erythrocyte.

## PfHSP70s are the Guardians of the Parasite-Resident and Exported Proteome

*P. falciparum* has only six Hsp70s (PfHsp70s; Shonhai et al., 2007; Shonhai, 2021) and four Hsp90s (PfHsp90s; Shahinas and Pillai, 2021). However, there is a highly expanded complement of JDPs, with 49 members (PfJDPs; Botha et al., 2007; Njunge et al., 2013; Pesce and Blatch, 2014; Dutta et al., 2021a).

All six PfHsp70s appear to be finely tuned to the malaria parasite lifecycle, playing an important role in parasite survival and virulence (Przyborski et al., 2015), with most being essential (Zhang et al., 2018), and a number of them shown to be inhibited by small molecules with anti-malarial activity (Chiang et al., 2009; Cockburn et al., 2011; Cockburn et al., 2014; Zininga et al. 2017a; Zininga et al. 2017b; Zininga et al. 2017c). The canonical and highly abundant cytoplasmic and nuclear *P. falciparum* Hsp70-1 (PfHsp70-1; Kumar et al., 1991; Pesce et al., 2008) has been shown to be essential (Zhang et al., 2018). PfHsp70-1 is regulated by a number of co-chaperones, including JDPs which deliver specialized substrates (Pesce et al., 2008; Botha et al., 2011; Njunge et al., 2015), *P. falciparum* Hsp70/Hsp90 organizing protein which enables transfer of substrates to PfHsp90 (PfHop; Gitau et al., 2012; Zininga et al., 2015), and the cytosolic Hsp70-like protein, PfHsp70-z (an Hsp110), serving as a nucleotide exchange factor (Zininga et al., 2016). The cytoplasmic PfHsp70-z is also essential (Muralidharan et al., 2012; Zhang et al., 2018), which may well be due to its highly effective protein aggregation suppression activity (Zininga et al., 2016). PfHsp70-1 has also been shown to have high ATPase activity (Matambo et al., 2004; Misra and Ramachandran, 2009; Makumire et al., 2021) and strong aggregation suppression activity (Botha et al., 2011), suggesting that it is a superior chaperone compared to human Hsp70s (Anas et al., 2020). Overall, the evidence suggests that PfHsp70-1 and PfHsp70-z are major players in proteostasis of the parasite cytoplasm.

In the endoplasmic reticulum (ER), there are two Hsp70s, PfHsp70-2 (a BiP/Grp78 homologue) and PfHsp70-y (a Hsp110/Grp170 homologue). PfHsp70-2 is essential (Zhang et al., 2018), and has been proposed to be involved in protein translocation into the ER, working with a PfJDP (PfSec63) and the PfSec translocon (Tuteja, 2007; Blatch and Zimmermann, 2009; Cortés et al., 2020; Shonhai, 2021). PfHsp70-2 was found to functionally associate with another PfJDP (Pfj2) and a protein disulfide isomerase (PDI-8) in the oxidative folding of ER proteins (Cobb et al., 2017). PfHsp70-y has also been shown to be essential, and to potentially interact with, and serve as a nucleotide exchange factor for PfHsp70-2, analogous to the cytoplasmic PfHsp70-z-PfHsp70-1 interaction (Zhang et al., 2018; Kudyba et al., 2019). Overall, the data suggest that these chaperones play an important role in protein quality control and proteostasis within the ER.

Very little is known about the proposed mitochondrial PfHsp70-3, except for the observation that PfHsp70-3 interacted with at least two N-rich malarial antigens (LaCount et al., 2005). Like PfHsp70-3, other PfHsp70s (PfHsp70-1, PfHsp70-z, and PfHsp70-x) have been reported to exhibit a preference for N-rich substrates (Muralidharan et al., 2012; Mabate et al., 2018; Lebepe et al., 2020; Rajapandi, 2020). Therefore, there is growing evidence that PfHsp70s may be finely tuned for the protection of unstable N-rich *P. falciparum* proteins from misfolding and aggregation.

PfHsp70-x is the only exported member of the PfHsp70 family, and has been shown to be localized to the PV and erythrocyte cytosol where it is found free or associated with PfJDPs (PFE0055c and PFA0660w) in mobile lipid containing complexes called J-Dots (Külzer et al., 2012; Grover et al., 2013; Behl and Mishra. 2019). Interestingly, PfHsp70-x is not essential; however, knockout compromised virulence (Charnaud et al., 2017), while knockdown compromised growth under stressful conditions similar to febrile episodes (Day et al., 2019). Recently, the crystal structures of the ATPase (Day et al., 2019) and substrate binding domains (Schmidt and Vakonakis, 2020) of PfHsp70-x were elucidated. Interestingly, PfHsp70-x contains an N-terminal signal sequence for secretion through the ER, but not the *Plasmodium* export element (PEXEL; Marti et al., 2004; Hiller et al., 2004), which has been shown to be required for the export of many *P. falciparum* proteins through the *Plasmodium* translocon of exported proteins (PTEX; de Koning-Ward et al., 2009; Beck et al., 2014; Elsworth et al., 2014; Elsworth et al., 2016). PfHsp70-x, like certain other PEXEL-negative *P. falciparum* proteins (PNEPs), is also successfully exported through the PTEX translocon (Rhiel et al., 2016). We are yet to elucidate exactly how proteins synthesized off the ribosome in the parasite cytosol, are threaded through the ER, across the plasma membrane, through the PV and the PV membrane, and into the erythrocyte cytosol or *via* the Maurer’s Cleft to the membrane, where they are folded and begin functioning. However, there is some evidence emerging that suggests that PfHsp70-2 and PfHsp70-x (and potentially other chaperones/co-chaperones such as PfJDPs) may collaborate with the core threading machinery of PTEX, a class I AAA + ATPase (PfHsp101; Russo et al., 2010; Matthews et al., 2019) in the chaperoning of exported *P. falciparum* proteins. For example, it has been shown that PfHsp101 is localized to the ER and the PV (Russo et al., 2010), and is able to preferentially associate with certain PEXEL-containing proteins within these compartments (Gabriela et al., 2022). PfHsp70-2 has been shown to not only interact with proteins secreted into the PV, but also with exported proteins, including the main virulence factor *P. falciparum* erythrocyte membrane protein 1 (PfEMP1; Saridaki et al., 2008; Batinovic et al., 2017; Cortés et al., 2020). In addition, it has been reported that PfHsp70-x associates with PfHsp101 (Charnaud et al., 2017; Cobb et al., 2017; Zhang et al., 2018) and PfEMP1 (Külzer et al., 2012). Therefore, in addition to its role in protein quality control and proteostasis in the ER, PfHsp70-2 may be playing a major role in the protein trafficking of secreted proteins, and in collaboration with PfHsp70-x also involved in the trafficking of exported proteins. It is tempting to speculate that two protein trafficking and folding pathways exist for exported proteins: 1) a pathway for PEXEL-containing proteins with PfHsp101 being the main chaperone and PfHsp70-2 and PfHsp70-x being auxiliary chaperones; and 2) a pathway for PNEPs with PfHsp70-2 and PfHsp70-x being the main chaperones which hand over to PfHsp101 in the PV or as a component of PTEX.

## PfJDPs Harness the Chaperone Power of Parasite and Host HSP70s

PfJDPs are ubiquitous, expressed in all compartments of the parasite, in the PV and in the host cell (Dutta et al., 2021a). There is emerging evidence that they play an important role in protein trafficking, folding, assembly and protection from misfolding and aggregation under stressful conditions (Daniyan et al., 2016; Dutta et al., 2021a). The majority of the PfJDPs are essential (Maier et al., 2008; Zhang et al., 2018), and nearly half them are exported into the erythrocyte and play a crucial role in promoting parasite virulence (Dutta et al., 2021a).

A number of the parasite-resident PfJDPs, have been identified as potential co-chaperone of PfHsp70-1 (PfHsp40/PF14_0359, Botha et al., 2011, Anas et al., 2020; PFB0595w, Njunge et al., 2015; and Pfj4/PFL0565w, Pesce et al., 2008). Like PfHsp70-1, PfHsp40 is essential (Zhang et al., 2018), cytosolic, constitutively expressed, and upregulated under stressful conditions (Botha et al., 2011). While they are the homologous chaperone pair to the canonical cytosolic human Hsp70-JDP pair (HSPA1A-DNAJA1), there are subtle but critical structural, biochemical and functional differences, with the *P. falciparum* pair shown to be a more effective chaperone machine (Anas et al., 2020). Furthermore, PfHsp40 has been found to be farnesylated and palmitoylated, leading to membrane localization (Mathews et al., 2021). Notably, farnesyl-PfHsp40 may well be the essential isoform of this PfJDP, as inhibition of farnesylation significantly compromised survival of the parasite under stress conditions.

PfHsp70-PfJDP pairs have also been identified within the ER, and appear to play an important role in protein translocation (PfHsp70-2 and PfSec63/PF13_0102; Marapana et al., 2018) and protein folding and quality control within that compartment (PfHsp70-2 and Pfj2/PF11_0099; Cobb et al., 2017). At least one PfJDP has been shown to be secreted through the ER, and partially localized to the PV (PFF1415c; Khosh-Naucke et al., 2018). PFF1415c was found to be essential for growth of erythrocyte-stage parasites; however, its precise function is yet to be determined. Pfj1 (PFD0462w) is the only PfJDP reported to be localized to the apicoplast (Kumar et al., 2010), which was contrary to a previous report which proposed it was targeted to the mitochondrion (Watanabe, 1987). Pfj1 has an unusually long and unique C-terminal region, and has been proposed to be capable of binding to the apicoplast genome and play a role in DNA replication (Kumar et al., 2010). Interesting, Pfj1 has been shown to have a functional J domain (Nicoll et al., 2007), and therefore is likely to associate with a partner Hsp70; however, none of the PfHsp70s have been shown to localize to the apicoplast.

A number of recent reports suggest that the exported PfJDPs may serve as co-chaperones of not only the exported PfHsp70-x, but also the host Hsp70. As mentioned in the previous section, two of the exported PfJDPs (PFA0660w and PFE0055c) associate with PfHsp70-x in J-dots within the erythrocyte cytosol (Külzer et al., 2010; Külzer et al., 2012; Grover et al., 2013; Petersen et al., 2016), and have been shown to be co-chaperones of PfHsp70-x (Daniyan et al., 2016; Dutta et al., 2021b). It has been proposed that these J-dots play a role in the trafficking and folding of exported proteins (Külzer et al., 2012; Behl et al., 2019; Gabriela et al., 2022). Interestingly, one of the J-Dot PfJDPs, PFE0055c, was found to be essential (Zhang et al., 2018), while the other (PFA0660w) was not. However, functional disruption of PFA0660w was found to causes defects in knob formation and cytoadherence, with further genetic and biochemical studies suggesting that the role of PFA0660w in host cell modification involved host Hsp70 (Diehl et al., 2021; Table 1). This finding is consistent with a previous study using a yeast two-hybrid system, which reported an interaction between three exported PfJDPs (PFA0660w, PFE0055c, and PFB0090c) and human Hsp70 (Jha et al., 2017; Table 1). PFB0090c, also called knob-associated Hsp40 (KAHsp40; structurally similar to PFE0055c and PFA0660w), has been shown to interact with components of PTEX and knobs, and may be involved in the genesis of knob complexes (Acharya et al., 2012). It is well established that the knob protein complex does not contain PfHsp70-x, but rather, host chaperones (Hsp70, Hsp90, and Hop), and there is significant evidence that human Hsp70 is involved in the assembly of knob protein complexes (Banumathy et al., 2002; Alampalli et al., 2018). Hence, it is plausible that PFB0090c occurs in a common complex with human Hsp70, and potentially serves as its co-chaperone (Table 1).

**TABLE 1:** Exported PfJDPs shown to functionally associate with PfHsp70-x or human Hsp70.

PlasmoDB	Common name	Proposed function	Hsp70 partner	References
PFA0660w		Localized to J-Dots; Folding of exported proteins (e.g., PfEMP1)	PfHsp70-x; hHsp70	Külzer et al. (2010; 2012); Grover et al. (2013); Daniyan et al. (2016); Jha et al. (2017); Behl et al. (2019); Diehl et al. (2021)
PFE0055c		Localized to J-Dots; Folding of exported proteins (e.g., PfEMP1)	PfHsp70-x; hHsp70	Küzer et al. (2010; 2012); Jha et al. (2017); Dutta et al. (2021b)
PFB0090c	KAHsp40	Localized to knobs; Folding and assembly of knob complexes	hHsp70	Acharya et al. (2012); Jha et al. (2017)
PF110034	eCiJP	Localized to J-Dots and the erythrocyte cytoskeleton; Folding of exported proteins; Folding and assembly of the cytoskeleton	PfHsp70-x; hHsp70	Sahu et al. (2022)

The largest group of PfJDPs are those members containing a J domain with a corrupted HPD motif (so-called type IVs), most of which appear to be exported (Botha et al., 2007; Njunge et al., 2013; Daniyan and Blatch, 2017). In fact, there is evidence that a number of the exported type IV PfJDPs are essential for parasite survival (e.g., PFB0085c and PF14_0013; Zhang et al., 2018), required for growth or survival under febrile conditions [e.g., PFA0110w, the ring-infected erythrocyte surface antigen protein (RESA); Silva et al., 2005; Diez-Silva et al., 2012], or involved in pathogenesis (e.g., PF10_0381; knockout causes loss of knobs; Maier et al., 2008). Recently, an exported type IV PfJDP, called eCiJP/PF11_0034 (and a paralogue of PF10_0381), was found to localize to J-Dots, associate with the erythrocyte cytoskeleton, and to potentially interact with host Hsp70 (HSPA1A) (Sahu et al., 2022; Table 1).

## Conclusion

The malaria parasite has adapted to its pathological co-existence with the human host, enabling it to overcome the extreme physiological and cellular challenges that it faces, particularly during the erythrocytic stages of the life cycle. *P. falciparum* appears to have finely tuned its molecular chaperone machinery to be highly efficient, particularly its PfHsp70-PfJDP pairs, which are found in most compartments of the parasite-infected erythrocyte. Most of these *P. falciparum* PfHsp70-PfJDP partnerships appears to have evolved to efficiently protect the N-repeat-rich parasite proteome from the toxic effects of aggregation and misfolding. Furthermore, and perhaps as importantly, the parasite appears to have harnessed the host Hsp70 chaperone machinery to enable it to renovate the infected erythrocyte for its survival and pathology. The molecular details of this host-parasite interface represent an important frontier of future research endeavours.
